# A Rare Case of Collision Tumor at the Periampullary Region

**DOI:** 10.1155/crip/7777605

**Published:** 2025-12-22

**Authors:** Kshitija Kale, Vikram Chaudhari, Vikas Ostwal, Shailesh V. Shrikhande, Kedar Deodhar

**Affiliations:** ^1^ Department of Pathology, University of Iowa, Iowa, Iowa, USA, uiowa.edu; ^2^ Department of Pathology, Tata Memorial Hospital, Mumbai, India, tmc.gov.in; ^3^ Department of Surgical Oncology, Tata Memorial Centre, Mumbai, India, tmc.gov.in; ^4^ Department of Medical Oncology, Tata Memorial Hospital, Mumbai, India, tmc.gov.in; ^5^ Division of Cancer Surgery, Gastrointestinal and Hepato-Pancreatic-Biliary Service, Tata Memorial Centre, Mumbai, India, tmc.gov.in

## Abstract

Collision tumors are rare neoplasms composed of two histologically distinct components without a transitional zone. Only nine cases involving periampullary adenocarcinoma and neuroendocrine tumor (NET) have been reported. A 55‐year‐old male presented with recurrent fever and jaundice. Imaging and biopsy revealed a periampullary lesion diagnosed as tubule‐villous adenoma with high‐grade dysplasia. He underwent pylorus‐preserving pancreatoduodenectomy. Histology revealed moderately differentiated intestinal‐type adenocarcinoma (pT2N1) with regional lymph node metastasis. Additionally, a 2‐mm well‐differentiated NET was found 2.5 cm proximal to the primary tumor, also with nodal metastasis. Immunohistochemistry confirmed distinct lineages, fulfilling the criteria for a collision tumor. This case represents a rare periampullary collision tumor with both adenocarcinoma and NET components showing independent lymph node metastases. The absence of transitional zones rules out MiNEN. This report underscores the importance of thorough pathological assessment and adds to the limited literature on the behavior and prognosis of periampullary collision tumors.

## 1. Introduction

Pancreatoduodenectomy is a standard surgical approach that offers a chance of curative surgery for periampullary carcinomas which are carcinomas arising within 2 cm of the major papilla in the duodenum [1]. Neuroendocrine tumor (NET) of the small intestine is uncommon, with the duodenum being a relatively more frequent site compared to the jejunum or the ileum [1]. The concept of collision tumor dates to 1966 where Michalinos et al. described a case of gastric carcinoid and adenocarcinoma [2]. The collision tumor comprises two distinct pathologies in the same anatomical site with no mixed or transitional area in between [2–4]. To the best of our knowledge, only nine cases of collision tumor of adenocarcinoma and neuroendocrine tumor at the periampullary site have been described in the literature [5, 6]. We present a rare case of periampullary adenocarcinoma colliding with a well‐differentiated neuroendocrine tumor with each showing metastasis independently to the regional lymph nodes. The peculiarities of this case are its rarity at the periampullary site, the coexistence of two distinct malignancies without a transitional zone, the unexpected metastatic potential of a small well‐differentiated NET, and the independent nodal metastasis of both tumors, which set it apart from nearly all previously documented reports.

## 2. Case Report

**Figure 1:**
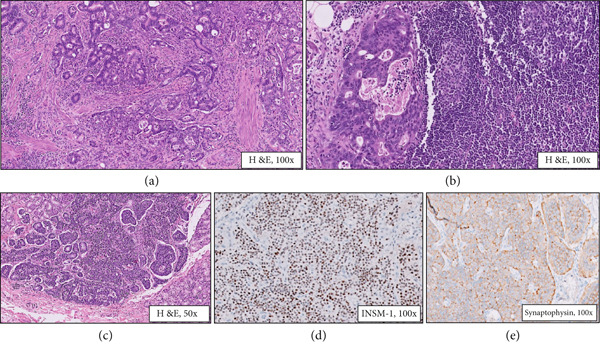
Histopathological and immunohistochemical features of the periampullary collision tumor. (a) Periampullary conventional adenocarcinoma (H&E, 100×). (b) Metastatic adenocarcinoma deposits in a regional lymph node (H&E, 100×). (c) Small focus of neuroendocrine tumor (2 mm) in the duodenal submucosa (H&E, 50×). (d) Nuclear positivity of neuroendocrine tumor cells for INSM‐1 (IHC, 100×). (e) Cytoplasmic positivity of neuroendocrine tumor cells for synaptophysin (IHC, 100×).

**Figure 2:**
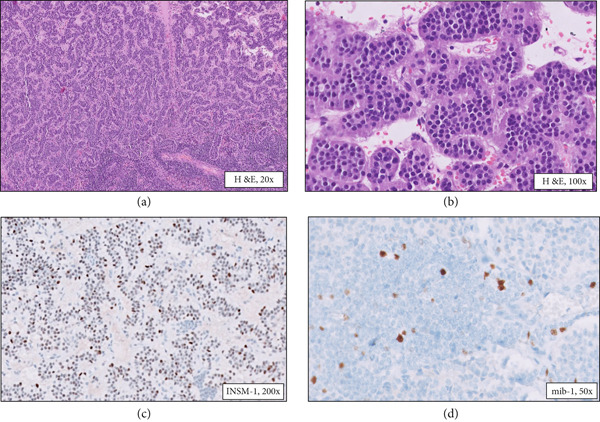
Nodal metastasis of the well‐differentiated neuroendocrine tumor. (a, b) Common hepatic lymph node showing metastatic neuroendocrine tumor (H&E, 20×). (c) Nuclear positivity of tumor cells for INSM‐1 (IHC). (d) Low proliferative activity of the tumor highlighted by MIB‐1 (Ki‐67) labelling index of 3%–4%.

A 55‐year‐old gentleman with complaints of recurrent fever with chills was evaluated for hyperbilirubinemia. Magnetic resonance cholangiopancreatography (MRCP) revealed a dilated terminal bile duct, and sphincterotomy was performed. However, the symptoms recurred 3 months later, this time with jaundice and a raised gamma glutamyl transferase. Endoscopic ultrasound‐guided biopsy was taken from the terminal common bile duct (CBD) which showed features of tubulovillous adenoma with high‐grade dysplasia. Serum tumor markers, CEA and CA 19‐9, were within the normal range. The surgical team planned for an open transduodenal ampullectomy for the patient. Intraoperatively, a 1.5‐cm firm tumor was identified involving the terminal CBD and ampullary region, thus proceeding with a pylorus‐preserving distal pancreatoduodenectomy. On gross examination of the specimen, a tumor was identified in the periampullary region. Adjacent duodenal mucosa appeared unremarkable. Sections from the periampullary tumor revealed a moderately differentiated adenocarcinoma, intestinal type (stage pT2) extending to the duodenal muscularis propria (Figure 1a) with metastases to two regional lymph nodes along the anterior surface of the pancreas (stage pN1) (Figure 1b). Immunohistochemistry revealed positive staining of tumor cells by CK‐7 (focal), CK‐20, and CDX‐2 indicating an intestinal type of adenocarcinoma. In addition, a small focus (2 mm) of well‐differentiated neuroendocrine tumor was identified in the submucosa of the adjacent duodenum, 2.5 cm proximal to the main tumor (Figure 1c). This focus showed nests and trabeculae composed of uniform cells. Immunohistochemistry for synaptophysin and INSM‐1 highlighted the tumor cells (Figure 1d,e). One of the four common hepatic lymph nodes was positive for metastasis from this neuroendocrine tumor (Figure 2a,b). The lymph node metastasis focus was tiny (1 mm) with no evidence of vascular emboli or atypical mitotic figures and was highlighted by INSM‐1 immunohistochemistry while the mib‐1 labeling index was 3%–5% (Figure 2c,d). Further sampling of the duodenum did not reveal any additional foci of neuroendocrine hyperplasia/tumor. The tumor was entirely evaluated under the microscope, and no other tumor focus was identified; also, there was no evidence of necrosis, vascular emboli, or perineural invasion.

In summary, the case showed the presence of a collision tumor at the periampullary region. Both components, adenocarcinoma and well‐differentiated neuroendocrine tumor showed independent metastasis to the regional lymph nodes, as confirmed by immunohistochemistry.

## 3. Discussion

When morphologically distinct tumors occur simultaneously at the same anatomical site, two distinct terminologies are applied. Collision tumors refer to two juxtaposed, but separate, tumor masses without histological transition between them, while composite tumors represent neoplasms of differing morphology but a common origin, with a recognizable transition zone [2–4, 7, 8]. In our case, adenocarcinoma and the well‐differentiated NET were identified 2.5‐cm apart, with intervening normal duodenal tissue and no evidence of transition or mixed morphology. Both components showed distinct and dissimilar histology, thereby fulfilling the definition of a collision tumor rather than a composite tumor. Other related entities include combined tumors, composed of histologically and immunophenotypically distinct yet intertwined cell populations of common origin, often sharing a driver mutation [9, 10]; multiple primary tumors, which arise at different sites or times [11]; and biphenotypic tumors, derived from a common progenitor with divergent differentiation but shared immunophenotypic features [12].

The World Health Organization (WHO) recognizes a separate entity, the mixed neuroendocrine–nonneuroendocrine neoplasm (MiNEN). MiNENs are defined by the coexistence of neuroendocrine and nonneuroendocrine components, each constituting at least 30% of the tumor mass [1, 13]. The nonneuroendocrine component is most often adenocarcinoma, while the neuroendocrine component may range from well‐differentiated to poorly differentiated neuroendocrine carcinomas. Although the 30% cutoff is somewhat arbitrary, it ensures that both components are biologically significant and not incidental findings. MiNENs are thought to arise either from pluripotent progenitor cells with divergent differentiation or through clonal evolution in which one tumor acquires additional alterations leading to a second lineage [1, 2, 13]. Clinically, prognosis is often dictated by the more aggressive component, usually the neuroendocrine element, and treatment is frequently tailored accordingly [2]. In contrast, our case did not fulfill criteria for MiNEN, given the small size of the NET, the absence of intermingling components, and the distinct separation of the tumors.

A review of the literature highlights the extreme rarity of periampullary collision tumors. The earliest report by Stamm et al. in 1986 described an ampullary carcinoid colliding with a duodenal adenocarcinoma [14]. Williams and colleagues later reported adenocarcinoma and NET with metastasis of both components to regional lymph nodes [15]. Marco et al. in 2007 documented an adenocarcinoma of the pancreatic head with an incidental duodenal carcinoid; lymph node metastasis was absent, and the patient succumbed 14 months after surgery [16]. A year later, a Taiwanese group reported large‐cell neuroendocrine carcinoma of the ampulla with a synchronous duodenal adenocarcinoma [17]. Niu et al. in 2010 presented a survival analysis of pancreatic and periampullary collision carcinomas, reporting three periampullary cases with a mean survival of 10 months, though none demonstrated dual metastasis [18]. Only one prior case has been reported in the Indian literature, describing a periampullary large‐cell neuroendocrine carcinoma colliding with signet‐ring cell adenocarcinoma, with metastasis limited to the neuroendocrine component [6]

Our case is distinctive in several respects. Both the adenocarcinoma and the well‐differentiated NET in our patient demonstrated independent nodal metastases, a finding that has not been previously described. Remarkably, the NET focus was only 2 mm in size, yet was capable of producing lymph node metastasis, highlighting the unpredictable behavior of even small NETs. Preoperative biopsy underestimated the pathology, revealing only tubulovillous adenoma with high‐grade dysplasia and failing to detect the dual malignancy, which underscores the limitations of small endoscopic samples in these cases. Furthermore, the patient′s initial presentation was atypical, with fever and chills rather than the classical obstructive features of periampullary malignancy, which may have contributed to diagnostic delay.

The pathogenesis of collision tumors remains speculative and poorly understood. Niu et al. proposed the role of an unidentified carcinogenic stimulus acting concurrently on different cell populations, resulting in the emergence of two independent neoplasms [18]. Another hypothesis suggests that one tumor may induce localized immunosuppression, creating a permissive environment for the evolution of a second tumor. While intriguing, these mechanisms remain unproven, and systematic molecular studies are lacking.

Although collision tumors are rare, the increasing number of case reports in recent years underscores the importance of recognizing this entity and maintaining vigilance for its occurrence. Reported examples include a colonic adenocarcinoma with large‐cell neuroendocrine carcinoma and gingival metastasis [19]; a primary pulmonary collision tumor comprising mixed squamous cell and glandular papilloma with a glomus tumor [20]; and metastasis of melanoma into an intracranial meningioma [21]. Other reported sites include the pineal region, featuring a pineocytoma and a pilocytic astrocytoma [22]. While several cases of combined mucinous neoplasms and granulosa cell tumors have been reported, only one collision tumor composed of a Sertoli–Leydig cell tumor and high‐grade serous carcinoma has been documented so far [23]. The advent of molecular pathology has opened new avenues for identifying collision tumors. For instance, Maryjka et al. described a gastroesophageal junction collision tumor in which the esophageal component demonstrated mismatch repair deficiency with loss of MLH1 and PMS2, whereas the gastric component exhibited distinct, nonoverlapping molecular alterations, including an *EML4::ALK* fusion and intact mismatch repair status, confirming two independent neoplasms [24].

In conclusion, our case highlights a rare periampullary collision tumor with highly unusual features, including independent metastasis from both adenocarcinoma and a minute well‐differentiated NET, distinct separation of the tumors, atypical clinical presentation, and diagnostic underestimation on preoperative biopsy. Such cases emphasize the biological unpredictability of collision tumors, the importance of thorough histopathological and immunohistochemical evaluation, and the need for documenting additional cases to improve understanding of their tumorigenesis, prognostic implications, and management.

## 4. Take Home Message

1. Collision tumors are rare entities characterized by the coexistence of two histologically distinct neoplasms in the same anatomical site without any transitional zone.
2. Periampullary collision tumors involving adenocarcinoma and NET are extremely rare, with only nine previously reported cases in the literature.
3. This case is unique as both the adenocarcinoma and well‐differentiated NET demonstrated independent lymph node metastases, highlighting their distinct biological behaviors.
4. Accurate diagnosis requires meticulous pathological examination and immunohistochemical profiling to distinguish between collision tumors, MiNEN, and composite tumors. Recognition of such rare entities is crucial for understanding tumorigenesis, guiding prognosis, and informing individualized therapeutic approaches.

## Conflicts of Interest

The authors declare no conflicts of interest.

## Funding

No funding was received for this manuscript.

## Supporting information


**Supporting Information** Additional supporting information can be found online in the Supporting Information section. CARE Checklist attached.

## Data Availability

Data sharing is not applicable to this article as no datasets were generated or analyzed during the current study.
